# Effect of Depression on Infertility in Traditional Iranian Medicine

**DOI:** 10.31661/gmj.v13i.3139

**Published:** 2024-03-11

**Authors:** Mohammadreza Mirzaei, Parinaz Kalejahi, Reza Mohammadinasab

**Affiliations:** ^1^ Department of Persian Medicine, Faculty of Traditional Medicine, Tabriz University of Medical Sciences, Tabriz, Iran; ^2^ Research Center of Psychiatry and Behavioral Sciences, Tabriz University of Medical Sciences, Tabriz, Iran

**Keywords:** Traditional Iranian Medicine, Infertility, Depression

## Dear Editor,

Infertility is a medical condition that can be caused by many different reasons in both men and women [1]. On the other hand, Infertility treatment as a relatively common medical problem is associated with imposing high economic costs on the family and society [2]. Identifying the factors that can affect the treatment of this disorder can be considered a suitable and less costly solution. In modern medicine, it has been stated that the diagnosis of infertility in couples can lead to psychiatric disorders. According to some studies, 20 to 60 percent of infertile patients suffer from mild to severe symptoms of depression and anxiety [3].

Traces of the theories of traditional Iranian physicians in the Middle Ages can be found in modern medicine today. These physicians collected the medical information available in Greece, Egypt, India, and China, added their knowledge, and presented theories that can be cited even today [4][5].

According to traditional Iranian medicine, infertility can stem from a multitude of factors, and the factors that contribute to decreased fertility are notably diverse in the context of Iranian medicine [6]. A very important point in the sources of traditional medicine in this regard is that from the perspective of Iranian traditional medicine, psychiatric disorders such as depression and anxiety can be effective factors in infertility and reduce fertility. Ibn Sina and other doctors of Iranian traditional medicine, including Razi and Ibn Abbas Ahwazi, consider depression and anxiety to be important factors in reducing fertility [6][7].

In traditional Iranian medicine, it is believed that depression with changes in temperament (according to the definition in the book of Khwarazmshahi collection, temperament is created by the combination of four elements (including water, wind, earth, and fire) could have a negative effect on brain chemistry and functions, which is involved in infertility [8]. This issue can be justified and debated in modern medicine. Several mechanisms are implicated in this process, with dopamine neurotransmitters being a noteworthy example [9].

In depressed patients, there is a disturbance in the dopamine system and low levels of dopamine have been linked to some important symptoms of depression such as apathy and feelings of hopelessness. On the other hand, treatment with dopamine agonists in infertility as a suppressor of prolactin from the pituitary gland has always been of interest [9][10].

An additional critical determinant is the existence of oxidative stress as a substantiated discovery within individuals experiencing a state of depression. Defective sperm function can be attributed to DNA damage, largely resulting from oxidative stress. [11].

According to the stated content, it can be concluded that the treatment of depression as a systemic disorder can have positive results in the treatment of infertility patients.

## Conflict of Interest

None.
